# The Impact of Urbanization and Human Mobility on Seasonal Influenza in Northern China

**DOI:** 10.3390/v14112563

**Published:** 2022-11-19

**Authors:** Jiao Yang, Xudong Guo, Ting Zhang, Qing Wang, Xingxing Zhang, Jin Yang, Shengjie Lai, Luzhao Feng, Weizhong Yang

**Affiliations:** 1 School of Population Medicine and Public Health, Chinese Academy of Medical Science (CAMS)/Peking Union Medical College (PUMC), Beijing 100730, China; 2 Department of Automation, Tsinghua University, Beijing 100084, China; 3 WorldPop, School of Geography and Environmental Science, University of Southampton, Southampton SO171BJ, UK

**Keywords:** seasonal influenza, human mobility, driver, China

## Abstract

The intensity of influenza epidemics varies significantly from year to year among regions with similar climatic conditions and populations. However, the underlying mechanisms of the temporal and spatial variations remain unclear. We investigated the impact of urbanization and public transportation size on influenza activity. We used 6-year weekly provincial-level surveillance data of influenza-like disease incidence (ILI) and viral activity in northern China. We derived the transmission potential of influenza for each epidemic season using the susceptible–exposed–infectious–removed–susceptible (SEIRS) model and estimated the transmissibility in the peak period via the instantaneous reproduction number (*R_t_*). Public transport was found to explain approximately 28% of the variance in the seasonal transmission potential. Urbanization and public transportation size explained approximately 10% and 21% of the variance in maximum *R_t_* in the peak period, respectively. For the mean *R_t_* during the peak period, urbanization and public transportation accounted for 9% and 16% of the variance in *R_t_*, respectively. Our results indicated that the differences in the intensity of influenza epidemics among the northern provinces of China were partially driven by urbanization and public transport size. These findings are beneficial for predicting influenza intensity and developing preparedness strategies for the early stages of epidemics.

## 1. Introduction

In temperate regions, the peak influenza season occurs in the winter months [1], and the scale of seasonal influenza epidemics can vary greatly between provinces and years [2,3]. However, little is known about the drivers of this variation. A better understanding of the factors that govern epidemic intensity is necessary for the public health system to accurately and promptly prepare for seasonal influenza epidemics.

Climatic factors are important drivers of influenza epidemics in temperate regions. Experimental studies have shown that a reduction in relative humidity improves the viability and transmission of influenza virus aerosols [4,5]. Epidemiological evidence also indicates that a reduction in relative humidity is associated with a higher risk of influenza A in the population [6]. Urbanization and human mobility are also believed to be drivers of influenza epidemics [7,8,9]. A simulation-based investigation in Australia highlighted that the increased peak prevalence and faster spreading rate of influenza pandemics could partially be attributed to an increase in population fractions living in cities [7]. A study of weekly incidence data from the United States found that the size of the urban population was positively associated with the incidence of city-level influenza and further showed that the intensity of influenza epidemics was shaped by urbanization and humidity [10]. Empirical evidence revealed that airline volume was a significant predictor of the spread of influenza between regions [8], and high mobility within countries (internal commuting) could accelerate epidemics [9]. However, these studies focused mainly on the impact of human mobility on interregional influenza epidemics. Evidence regarding the influence of human mobility on intracity or intraprovincial epidemics is limited.

Recent studies on influenza epidemics have revealed unexplained differences between provinces with similar urbanization and climate conditions in China [2,3,11], suggesting that there are other unidentified factors driving the differences in influenza epidemics between provinces. China is a vast country that comprises provinces with different climatic and economic backgrounds. These factors have led to varying levels of heterogeneity regarding population structure and mobility. Therefore, we assumed that the unexplained interprovince differences in influenza epidemic intensity may be caused by the heterogeneity of population mobility in provinces with similar climates and urbanization levels. Higher human mobility inside a province increases close contact between people, and thus the transmission of the influenza virus among people may be enhanced.

In the present study, we explored the above hypothesis by using 6 years (2012 to 2017) of data on weekly influenza-like disease and virus activity in 14 provinces in northern China.

## 2. Methods 

### 2.1. Data

The temperature of both the environment and the dew point for each province were obtained from the China Meteorological Administration to calculate the relative humidity, using the R package ‘humidity’ (R software, version 4.2.1). The approximating function can closely simulate relative humidity: $r\left(t\right)=u\times cos\times \left(2\times \pi \left(t-5\right)\right)/52+m$. Census data, including population size, urbanization, and public transportation data, were recovered from the China National Bureau of Statistics [12]. Weekly influenza-like disease incidence rate data (ILI) and viral detection positive rate data for each province were obtained from the Chinese National Influenza Surveillance Network. Referring to previous studies [13,14], proxy measures of the weekly incidence rate (referred to as the ‘incidence rate’) were obtained by multiplying the ILI percentage among patients visiting sentinel hospitals with the proportions of influenza-positive specimens. This proxy is considered a precise representation of the activity of influenza infection [15,16].

### 2.2. SEIRS Model

Referring to previous studies [3,10], we constructed a susceptible–exposed–infectious–removed–susceptible (SEIRS) compartmental model to work with province-level weekly incidence rate data (the ILI rate × the proportions of influenza-positive specimens). Susceptible (*S*) refers to individuals at risk of infection with influenza, representing approximately 90% of the total population. Exposed (*E*) refers to people in the latent period. Infectious (*I*) refers to people who have been infected. Removed (*R*) refers to people who have recovered or died. The SEIRS model consists of the following ordinary differential equations:

$$dS/dt=-N^{-1}\beta SI+\delta \left(N-S-E-I\right)$$

$$dE/dt=N^{-1}\beta SI-\varepsilon E$$

dI/dt=εE−γI

dR/dt=γI

N=S+E+I+R

where δ is the rate of reinfection, which is equal to 1/52; *ε* is the rate of infection after exposure, which is equal to 7; and *γ* is the rate of recovery from infection, which is equal to 7/2. The values of δ, ε, and γ were taken from Dalziel’s research [10]. The generation time was assumed to be 3 days.

After a certain period [17,18], the immunity of infected individuals weakens and these individuals enter the susceptible compartment. New infections are generated when a susceptible individual comes into contact with an infected individual at a rate of *βSI/N*, where *N* refers to the size of the population. In a stable population, the incidence of new infections is governed by the transmission function $\beta (t)=g+\sigma^{-\omega r\left(t\right)}$*,* where g refers to the maximum gain in the transmission potential at 0 relative humidity and ω refers to the rate of the loss of viral viability caused by relative humidity. The transmission function β(t) is composed of the sum of two parts: a seasonally invariant base transmission potential *g*, which refers to transmission between individuals under the same climatic conditions (in this case, the impact of climate is 0), and an additional transmission governed by relative humidity, $\sigma^{-\omega r\left(t\right)}$, which increases with the decrease in relative humidity in Chinese provinces in winter and thus increases the risk of transmission between individuals under different climate conditions.

### 2.3. GLM Model

A generalized linear model (GLM) of the SEIRS model was further constructed to explore the patterns of influenza dynamics by fitting the incidence data. GLM avoids the defects associated with the nonlinearity of the SEIRS model. The corresponding generalized linear model is as follows:

$$Y_{n+1,j}=a\cdot W_{nj}+b\cdot X_{nj}r_{nj}+c\cdot X_{nj}P_{nj}+Q_{nj}$$

where *Y_nj_* = *log*[*I_nj_*], *I_nj_* indicate the number infected in week *n* of season *j*. We obtained *I_nj_* by multiplying the incidence rate (the ILI rate × the proportions of influenza-positive specimens) with the province population size. The parameter vector a is given by $a=\left[log\left(g\right),log\left(g+\sigma_{1}\right),\ldots ,log\left(g+\sigma_{6}\right)\right]$*,* which is estimated from the SEIRS model. The design vector *W_nj_* with seven elements indicates whether the data point associated with (*n*,*j*) is in the off-peak regime or in one of the six influenza seasons. *b* and *c* are parameter vectors with two elements, *b* = [*ω*_1_, *ω*_2_] and *c* = [*ρ*_1_, *ρ*_2_], where *b* is obtained by fitting the relationship between relative humidity and viral viability and *c* is obtained from the observed incidence data. *X_nj_* is a design vector with two elements that indicate whether the point associated with (*n*,*j*) is in the off-peak or peak influenza season. $P_{nj}$ indicates cumulative incidence, $P_{nj}=\frac{1}{N}\Sigma_{k=0}^{n}I_{kj}$. *O_nj_* is an offset term *O_nj_* = *log*(<*S*_0*j*_>) − *log*(*N*) + *αY_nj_*, where <*S*_0*j*_> = 0.9*N* refers to the expected population-level initial susceptibility each season, taken from Wang and colleagues’ study [19]. The influenza peak was defined as extending from 5 weeks before the peak incidence rate observed in each season to 5 weeks after [20]. A detailed explanation of the GLM model was provided in a previous study [10].

### 2.4. Estimation of Transmissibility

The weekly instantaneous reproduction number $R_{t}$ was estimated according to the Bayesian framework applied to the branching process model proposed by Cori et al. [21], which is an extension of Fraser method [22]. Fraser proposed that the renewal estimation equation for the $R_{t}$ of an epidemic could be written as:

(1) $$R_{t}=\frac{I_{t}}{\sum_{s=0}^{m}w_{s}I_{t-s}}$$

where $I_{t}$ refers to the number of reported cases (here, the incidence rate times a constant) between time t and time t+1, and $w_{s}$ refers to the generation time distribution, such that $\overset{m}{\underset{s=0}{\sum }}w_{s}=1$. The expected incidence at time t is Poisson distributed with a mean ($R_{t}\overset{m}{\underset{s=0}{\sum }}w_{s}I_{t-s}$). The transmissibility is assumed to be constant over the time period [t−τ, t] and measured by $R_{\left[t-\tau ,t\right]}$; then, the likelihood of $I_{t-\tau },\;\ldots \;\ldots \;\ldots ,\;I_{t}$ given the reproduction number $R_{\left[t-\tau ,t\right]}$ and $I_{0},\;\ldots \;\ldots \;\ldots ,\;I_{t-\tau -1}$ is as follows:

(2) $$P(I_{t-\tau },\;\ldots \;\ldots \;\ldots ,\;I_{t}\left|I_{0},\;\ldots \;\ldots \;\ldots ,\;I_{t-\tau -1},\;w,\;R_{\left[t-\tau ,t\right]})\right.=\overset{t}{\underset{s=t-\tau }{\prod }}\frac{\;e^{-R_{\left[t-\tau ,t\right]}\Lambda_{s}}\;{\left(R_{\left[t-\tau ,t\right]}\Lambda_{s}\right)}^{I_{s}}}{I_{s}!}$$

where $\Lambda_{s}=\overset{m}{\underset{s=0}{\sum }}w_{s}I_{t-s}$. The generation time distribution is a gamma distribution with a mean of 3 days (SD = 1.5 d) and is assumed to be constant throughout an epidemic. A Bayesian framework with a gamma-distributed prior with parameters (a, b) was developed for $R_{\left[t-\tau ,t\right]}$, and the posterior joint distribution of $R_{\left[t-\tau ,t\right]}$ can be derived as proportional to

(3) $$R_{\left[t-\tau ,t\right]}\left(\mathrm{posterior}\right)=R_{\left[t-\tau ,t\right]}{}^{a+\overset{t}{\underset{s=t-\tau }{\sum }}I_{s}-1}{\;e}^{-{\;R}_{\left[t-\tau ,t\right]}\left(\overset{t}{\underset{s=t-\tau }{\sum }}\Lambda_{s}+\frac{1}{b}\right)}\;\overset{t}{\underset{s=t-\tau }{\prod }}\frac{\Lambda_{s}{}^{I_{s}}}{I_{s}!}$$

Equation (3) indicates that the posterior distribution of $R_{\left[t-\tau ,t\right]}$ is a gamma distribution with the parameters $(a+\overset{t}{\underset{s=t-\tau }{\sum }}I_{s},({\overset{t}{\underset{s=t-\tau }{\sum }}\Lambda_{s}+\frac{1}{b})}^{-1})$.

### 2.5. Regression Analysis with Transmissibility β and R_t_ of Each Influenza Season

A simple linear regression model was used to explore the relationship between driving factors and β. R-squared values (R^2^) were used to quantify the impact of individual drivers. To make the results more intuitive, we used the same method to further quantify the relationship between each driving factor and the simulated $R_{t}$. Because public transportation is easily affected by population size and urbanization, for example, in provinces with larger population sizes and higher urbanization, the accessibility of public transport is higher and more people may use public transportation. Therefore, we can more accurately represent human mobility per unit density. A combined mobility index, *h,* was calculated using population size (*PS*), urbanization (*U*), and public transportation (*PT*) to represent human mobility more accurately: *h* = $\log\left(\frac{PS*U}{PT}\right)$. A higher value of *h* indicates more frequent population mobility. Differences in Akaike information criteria (ΔAIC) were used to estimate the relative quality of the GLM, where higher values indicate models with poorer relative support.

## 3. Results

**Figure 1:**
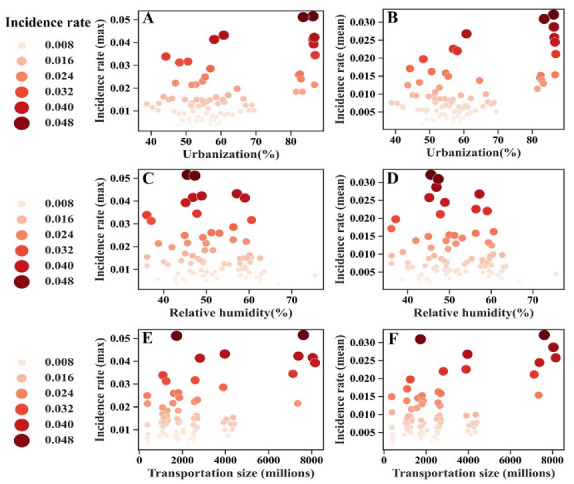
Bubble charts demonstrating the incidence rate in provinces with different levels of urbanization, transport, and relative humidity (**A**–**F**). Provinces with higher max and mean incidence tended to have a higher magnitude of urbanization (**A**,**B**), lower relative humidity (**C**,**D**), and larger transportation size (**E**,**F**).

Annual data on population size, urbanization, and public transportation size are presented in Table 1. As shown in Figure 1, influenza incidence varies with urbanization, climate, and transportation size. The maximum and mean incidence of influenza at peak times tended to be higher in provinces with larger magnitudes of urbanization and larger transportation sizes (Figure 1A–D).

**Figure 2:**
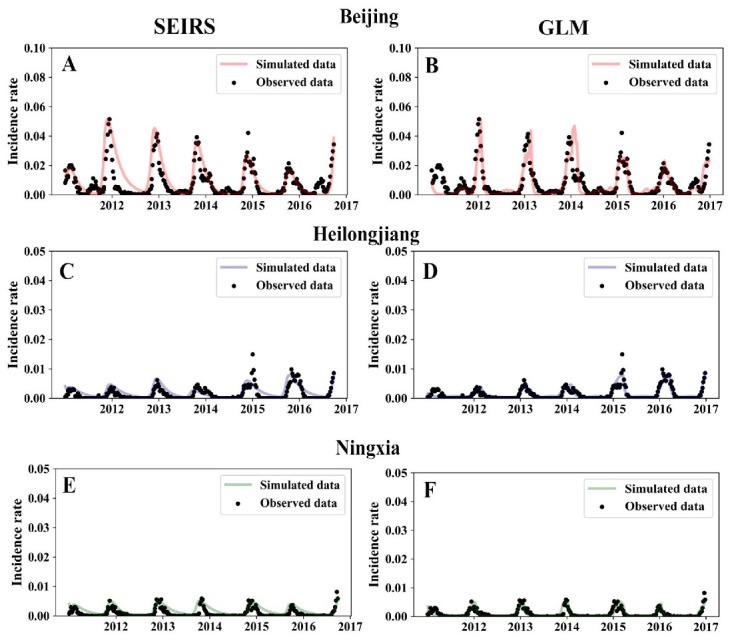
Transmission potential and relative humidity predicted the observed differences in the intensity of the influenza epidemics in northern Chinese provinces (**A**–**F**). (**A**,**C**,**E**) simulated results of the SEIRS model in three provinces. (**B**,**D**,**F**) fitted results of the GLM model in three provinces.

**Figure 3:**
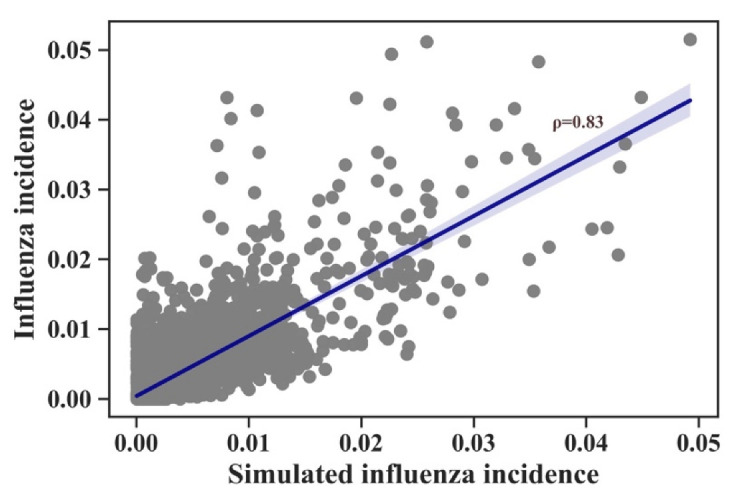
Observed versus simulated influenza incidence in all provinces. Gray points show simulated influenza incidence rate. Blue line shows the fitted line between simulated influenza incidence and observed incidence.

The SEIRS model was used to fit the influenza incidence rate data to explore the reasons for the temporal and spatial differences in the intensity of the influenza epidemics. As described in the Methods section, the transmission potential of each influenza season in each province could be obtained using the SEIRS model. Influenza epidemics vary in intensity by year and province, indicating a difference in transmission potential. The SEIRS model is a common method for fitting influenza time series data. However, the model is nonlinear; thus, minor changes in input parameters can cause significant changes in the prediction results. Therefore, a general function of the SEIRS model was constructed to work with province-level influenza incidence data. The results are shown in Figure 2. Ten fitted parameters (Supplementary Table S1) were obtained using province-level time series models. The results were obtained for the following three provinces randomly selected from the total of 14: Beijing, Heilongjiang, and Ningxia. Spearman’s r = 0.83 for the comparison of the observed and predicted influenza incidence (Figure 3). 

**Figure 4:**
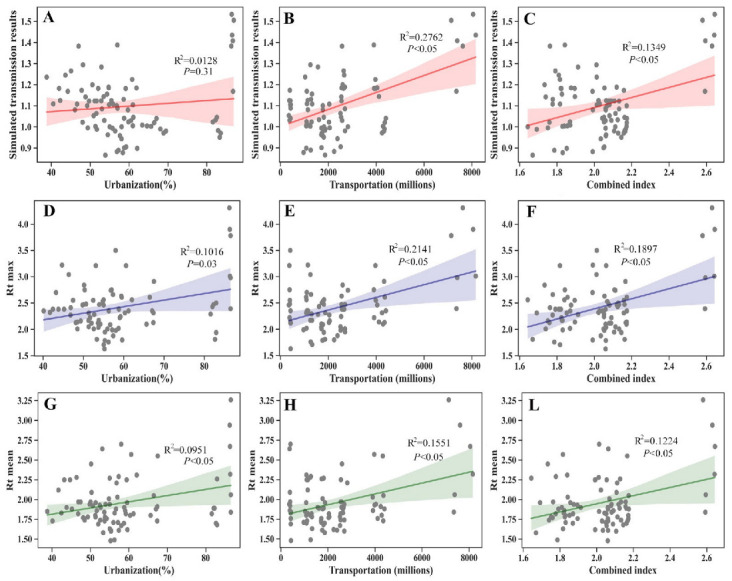
Urbanization, transportation size, and combined index estimated from census data predicted transmission potential, maximum *R_t_*, and mean *R_t_* during peak period of influenza season (**A**–**L**). Gray points show transmission potential, maximum *R_t_*, and mean *R_t_* during peak period of influenza season estimated from the influenza incidence rate. Red lines refer to the prediction for transmission potential during peak period of influenza season (**A**–**C**). Purple lines refer to the prediction for maximum *R_t_* during peak period of influenza season (**D**–**F**). Green lines refer to the prediction for mean *R_t_* during peak period of the influenza season (**G**–**L**).

The early transmission potential obtained by SEIRS is only a mathematical value, and its practical significance is limited. In this regard, we further explored the factors that influenced the transmission potential. As shown in Figure 4, urbanization and public transportation size could explain 1.28% and 27.62% of the variation in the annual transmission potential, respectively. Close contact between individuals is a prerequisite for influenza transmission. The frequency of close contact can directly affect the transmission potential of the influenza virus between persons. A larger public transport system does not necessarily mean that contact between persons is more frequent. To meet the commuting needs of residents, public transportation may be more extensive in areas with large populations. The size of public transportation per unit population in urban areas can reduce the impact of population size to better represent the transmission potential of contact between people. Urbanization, population size, and public transportation size were used to calculate the combined index *h*, which indicated population mobility. The results showed a positive correlation between the combined index *h* and the annual transmission potential, R^2^ = 0.1349 (*p* < 0.05).

In addition, the association between urbanization, public transportation size, and the combined index *h* was also examined with the maximum *R_t_* and mean *R_t_* at the peak of each influenza season. Urbanization and public transportation size were significant drivers of max *R_t_* and mean *R_t_* during the peak period of each influenza season.

## 4. Discussion

Weekly surveillance data on outpatient ILI and virus activity from 14 provinces in northern China revealed that the annual transmission potential was positively associated with the size of public transportation. The maximum *R_t_* and mean *R_t_* of each influenza season during the peak period estimated from province-level incidence data were positively correlated with urbanization and the size of public transportation. The results presented here suggest that, at least in northern China, the intensity of the influenza epidemic may be governed by urbanization and intra-city human mobility.

Climate conditions, urbanization, and human mobility play a significant role in the spread of seasonal influenza. Relative humidity is an important environmental factor that affects the survival of influenza viruses in aerosols, and it is also a crucial driving factor for influenza seasonality [23]. In the current study, we controlled for the influence of climate on transmission by fitting approximate functions. Therefore, the annual transmission potential refers to the comprehensive influence of other factors, excluding climate conditions.

The instantaneous reproduction number (*R_t_*) is typically used to characterize real-time transmissibility. A higher *R_t_* value indicates a higher transmission potential. The pathogen spreads when *R_t_* > 1 and is under control when *R_t_* < 1. We calculated the maximum *R_t_* and the mean *R_t_* of each influenza season for 14 provinces to quantify the transmissibility at peak times.

Further analysis showed that the size of public transport was positively correlated with the yearly transmission potential. This was consistent with the results of a previous simulation study [24]. Globally, people traveling by air cause the transmission of pandemic and seasonal influenza viruses, especially the A/H3N2 viruses [25,26,27,28,29,30]. At the regional scale, the spatial transmission of influenza is dominated by patterns of human contact, including school closure times and commute patterns [2,31,32]. In the current study, the maximum peak *R_t_* and the mean peak *R_t_* of the influenza season were positively associated with the size of public transport, which could explain the variations for more than one fifth of the maximum peak *R_t_* variations and about one fifth of the peak mean *R_t_*, respectively. These results provide new evidence for understanding the impact of human mobility on influenza epidemics.

Previous studies have examined the impact of urbanization on the intensity or epidemic patterns of influenza [2,7,10]. However, the definitions of urbanization vary between studies. For example, in Dalziel et al.’s study, urban population size is regarded as an indicator of urbanization [10]. In the studies by Lei and Zachreson, urbanization refers to the proportion of the total population living in urban areas [7,10]. In our study, different urbanization indicators were used to evaluate the relationship between urbanization and influenza transmission. Our results showed that the proportion of the total population living in urban areas was also positively correlated with the maximum peak *R_t_* and the mean peak *R_t_* of the influenza season. However, we did not find a consistent positive relationship between urban population density, urban population size, and influenza transmission (Supplementary Figures S2 and S3). Our findings suggest that the proportion of the total population living in urban areas may be a better indicator for studying the relationship between urbanization and influenza transmission in northern China compared with urban population size and urban population density. 

Two reasons may be responsible for this result. First, regarding infectious diseases, current explosive trends in urbanization mean that more people are concentrated in urban regions. Coupled with the spread of suburbs, this can lead to large hubs in the commuter interaction network, which can cause a faster spread of infectious diseases between work and home [33,34]. Second, public transportation (buses and subways) is a common means of traveling in many cities around the world; thus, if an infected person interacts closely with other users of public transportation on a bus or subway, combined with insufficient ventilation and overcrowded conditions, it can increase the risk of influenza for other uninfected people and lead to the spread of influenza among colleagues and family members [35].

A higher transmission potential and *R_t_* indicate that the number of infected cases will increase in a short period of time, requiring increased surge capacity in the public health system, including primary care facilities and clinical laboratories [36]. The significance of our study is that, when the influenza season arrives, it can help predict the intensity of the influenza epidemic according to urbanization and human mobility to prepare for its medical and social impact in advance. Additionally, obtaining information on transmissibility at peak times is beneficial for mitigating influenza spread by vaccination and taking non-pharmaceutical interventions (NPIs) in the early stages of epidemics [37,38].

A potential limitation of our study was that school holidays were not included in our model. Previous studies have emphasized the importance of children in the spread of influenza, and the impact of school holidays and school closures on transmissibility [16,39,40]. Additionally, we did not have information on the impact of antigen drift and host immunity on epidemics. However, a study based on the city-level analysis of the subtypes and antigenical characteristics of the influenza virus in Australia demonstrated that antigenic novelty has limited effects on epidemic size. It suggested that other factors drive influenza epidemics apart from host immunity at the local scale in temperate areas [41].

## 5. Conclusions

In conclusion, urbanization and human mobility were positively associated with the intensity of influenza. Increased commuting by public transport (including buses and subways) can accelerate the spread of influenza. Monitoring flows for public transport may be conducive to early detection and response to influenza epidemics.

## Figures and Tables

**Table 1:** Characteristics of the fourteen provincial-level administrative divisions during 2012–2017.

	Year	Province													
		Beijing	Tianjin	Hebei	Shanxi	Inner Mongolia	Liaoning	Jilin	Heilongjiang	Shandong	Henan	Shaanxi	Gansu	Qinghai	Ningxia
Population size (millions)	2012	20.69	14.13	72.88	36.11	24.90	43.89	27.50	38.34	96.85	94.06	37.53	25.78	5.73	6.47
	2013	21.15	14.72	73.33	36.30	24.98	43.90	27.51	38.35	97.33	94.13	37.64	25.82	5.78	6.54
	2014	21.52	15.17	73.84	36.48	25.05	43.91	27.52	38.33	97.89	94.36	37.75	25.91	5.83	6.62
	2015	21.71	15.47	74.25	36.64	25.11	43.82	27.53	38.12	98.47	94.80	37.93	26.00	5.88	6.68
	2016	21.73	15.62	74.70	36.82	25.20	43.78	27.33	37.99	99.47	95.32	38.13	26.10	5.93	6.75
	2017	21.71	15.57	75.20	37.02	25.29	43.69	27.17	37.89	100.06	95.59	38.35	26.26	5.98	6.82
Urbanization (%)	2012	86.20	81.55	46.80	51.26	57.14	65.65	53.70	59.60	52.43	42.43	50.02	38.75	47.44	50.67
	2013	86.30	82.01	48.12	52.66	58.71	66.45	54.20	57.40	53.75	43.80	51.31	40.13	48.51	52.01
	2014	86.35	82.27	49.33	53.79	59.51	67.05	54.81	58.01	55.01	45.20	52.57	41.68	49.78	53.61
	2015	86.50	82.64	51.33	55.03	60.30	67.35	55.31	58.80	57.01	46.85	53.92	43.19	50.30	55.23
	2016	86.50	82.93	53.32	56.21	61.19	67.37	55.97	59.20	59.02	48.50	55.34	44.69	51.63	56.29
	2017	86.50	82.93	55.01	57.34	62.02	67.49	56.65	59.40	60.58	50.16	56.79	46.39	53.07	57.98
Public transportation size (millions)	2012	7615.78	1299.51	2039.54	1248.38	963.49	4283.67	1705.61	2239.56	3982.68	2637.18	2545.99	1028.44	391.35	391.35
	2013	8047.75	1609.27	2027.27	1563.64	1086.85	4356.33	1713.78	2381.56	4113.11	2661.57	2507.32	1107.11	417.60	417.60
	2014	8158.48	1810.72	2053.42	1314.96	1070.99	4401.65	1768.67	2513.60	4038.54	2638.19	2692.46	1137.38	349.71	427.44
	2015	7383.84	1858.13	1872.08	1321.32	1076.74	4347.10	1754.52	2574.76	3900.26	2570.70	2692.03	1098.23	379.09	423.77
	2016	7349.53	1807.90	1860.48	1263.50	1145.88	4242.62	1713.69	2534.20	3911.36	2539.10	2694.06	1145.49	377.68	418.98
	2017	7133.96	1732.79	1818.29	1271.83	1083.90	4328.24	1772.18	2550.84	3967.77	2594.21	2817.40	1249.46	367.10	407.44

## Data Availability

Due to the potentially sensitive information included, the original dataset is not public and is available from the corresponding author upon reasonable request.

## Supplementary Materials

The following supporting information can be downloaded at: https://www.mdpi.com/article/10.3390/v14112563/s1. Figure S1: The map indicating the provinces studied in northern China. The color indicates the climatic domain: cold- temperate (black); mid-temperate (blue); warm-temperate (green); Table S1: Summary statistics on the means of fitted model parameters across provinces; Figure S2: The association for urban population density with transmission potential (A), maximum *R_t_* (B) and mean *R_t_* (C) in peak time of influenza season; Figure S3: The association for urban population size with transmission potential (A), maximum *R_t_* (B) and mean *R_t_* (C) in peak time of influenza season.
