# Supplementary material for: The Impact of Urbanization and Human Mobility on Seasonal Influenza in Northern China
Source: Viruses. 2022 Nov 19;14(11):2563. doi: 10.3390/v14112563 (PMC9697484; doi:10.3390/v14112563)
Supplement: Supplementary file 1 [file viruses-14-02563-s001.zip › viruses-2010277-supplementary.pdf]

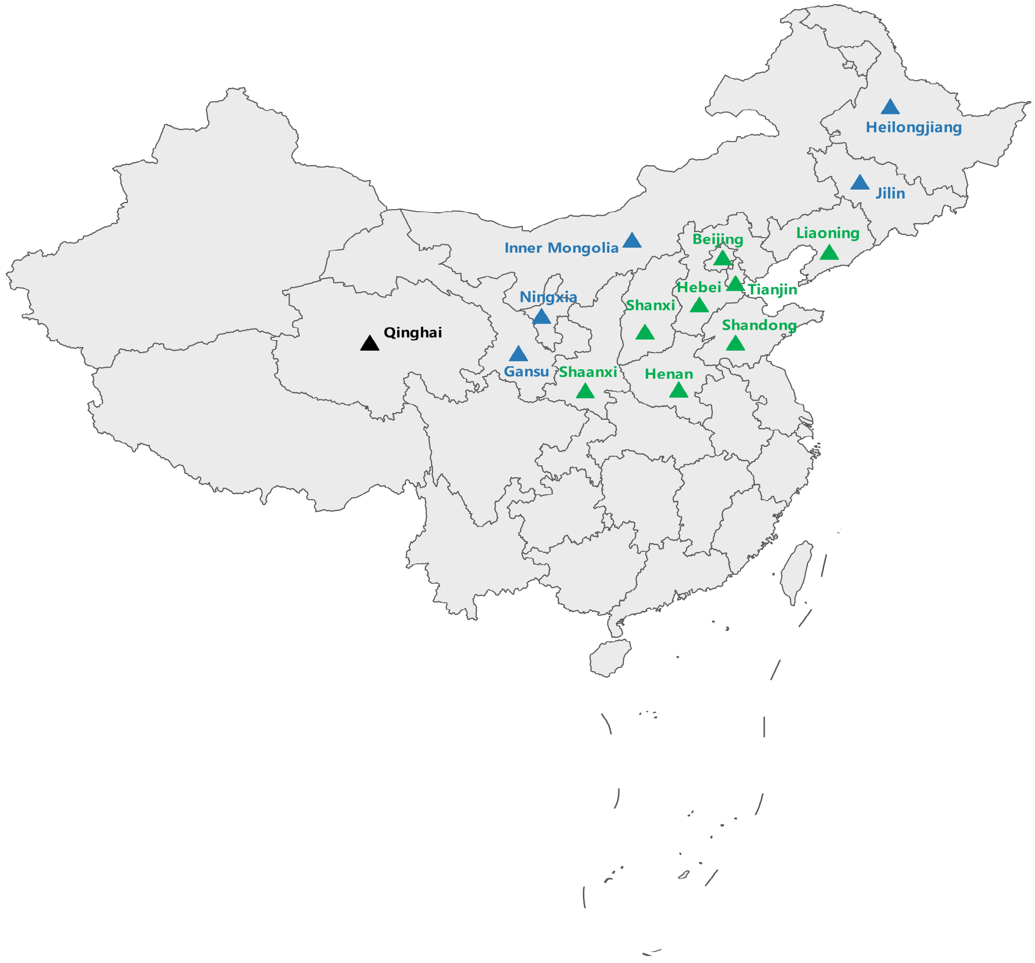

Figure S1. The map indicating the provinces studied in northern China. The color indicates the climatic domain: cold- temperate (black);,mid-temperate (blue); warm-temperate(green).

Table S1. Summary statistics on the means of fitted model parameters across provinces.

Parameter	Meaning	25th percentile	Median	Mean	75th percentile
σ_1	Transmission gain 12/13	2.73	3.05	3.00	3.26
σ_2	Transmission gain 13/14	2.78	2.95	3.09	3.12
σ_3	Transmission gain 14/15	2.78	2.95	3.05	3.12
σ_4	Transmission gain 15/16	2.69	2.99	3.11	3.25
σ_5	Transmission gain 16/17	2.60	2.77	2.78	2.92
σ_6	Transmission gain 17	2.72	3.04	3.12	3.31
ρ_1	Off-peak reporting (%)	0.00	0.00	0.00	0.00
ρ_2	Peak reporting (%)	1.56	2.42	2.78	3.94
ω_1	Off-peak loss rate	0.42	0.50	0.61	0.86
ω_2	Peak loss rate	0.42	0.53	0.79	0.86

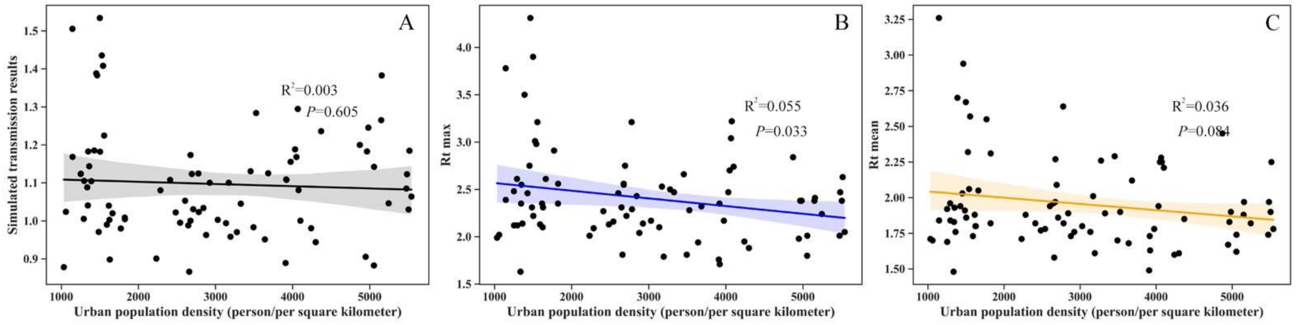

Figure S2. The association for urban population density with transmission potential (A), maximum R_t (B) and mean R_t (C)in peak time of influenza season.

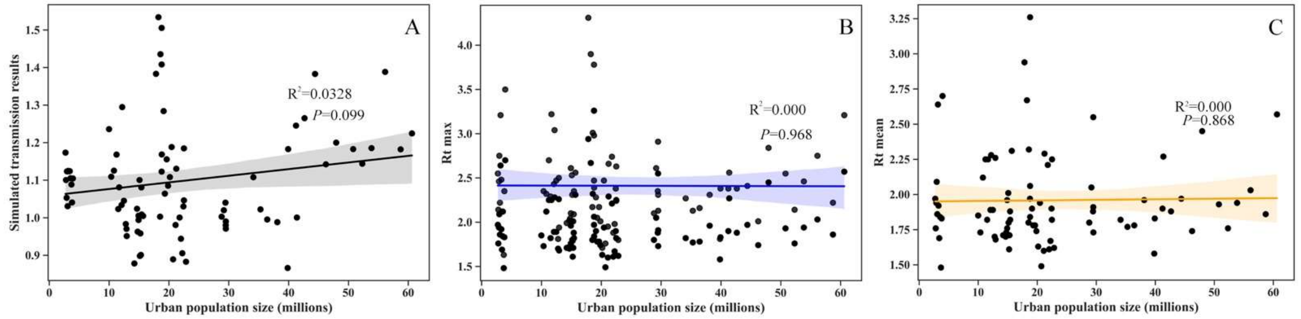

Figure S3. The association for urban population size with transmission potential (A), maximum R_t (B) and mean R_t (C)in peak time of influenza season.
